# Development and validation of nomogram prediction model for severe kidney disease in children with Henoch–Schönlein purpura: A prospective analysis of two independent cohorts—forecast severe kidney disease outcome in 2,480 hospitalized Henoch–Schönlein purpura children

**DOI:** 10.3389/fimmu.2022.999491

**Published:** 2022-10-12

**Authors:** Ke Wang, Xiaomei Sun, Shuolan Jing, Li Lin, Yao Cao, Xin Peng, Lina Qiao, Liqun Dong

**Affiliations:** ^1^ Division of Pediatric Pulmonology and Immunology, West China Second University Hospital, Sichuan University, Chengdu, China; ^2^ National Center for Birth Defects Monitoring of China, West China Second University Hospital, Sichuan University, Chengdu, China; ^3^ Key Laboratory of Birth Defects and Related Diseases of Women and Children (Sichuan University), Ministry of Education, West China Second University Hospital, Sichuan University, Chengdu, China; ^4^ National Health Council Key Laboratory of Chronobiology, Sichuan University, Chengdu, China

**Keywords:** severe kidney disease, Henoch–Schonlein purpura, cohorts, nomogram, prediction model

## Abstract

This study aimed to develop and validate a nomogram to forecast severe kidney disease (SKD) outcomes for hospitalized Henoch–Schönlein purpura (HSP) children. The predictive model was built based on a primary cohort that included 2,019 patients with HSP who were diagnosed between January 2009 and December 2013. Another cohort consisting of 461 patients between January 2014 and December 2016 was recruited for independent validation. Patients were followed up for 24 months in development/training and validation cohorts. The data were gathered at multiple time points after HSP (at 3, 6, 12, and 24 months) covering severe kidney disease as the severe outcome after HSP. The least absolute shrinkage and selection operator (LASSO) regression model was utilized to decrease data dimension and choose potentially relevant features, which included socioeconomic factors, clinical features, and treatments. Multivariate Cox proportional hazards analysis was employed to establish a novel nomogram. The performance of the nomogram was assessed on the aspects of its calibration, discrimination, and clinical usefulness. The nomogram comprised serious skin rash or digestive tract purpura, severe gastrointestinal (GI) manifestations, recurrent symptoms, and renal involvement as predictors of SKD, providing favorable calibration and discrimination in the training dataset with a C-index of 0.751 (95% CI, 0.734–0.769). Furthermore, it demonstrated receivable discrimination in the validation cohort, with a C-index of 0.714 (95% CI, 0.678–0.750). With the use of decision curve analysis, the nomogram was proven to be clinically useful. The nomogram independently predicted SKD in HSP and displayed favorable discrimination and calibration values. It could be convenient to promote the individualized prediction of SKD in patients with HSP.

## Introduction

Henoch–Schönlein purpura (HSP), also known as immunoglobulin A (IgA) vasculitis, is the most popular leukocytoclastic vasculitis, affecting the small vessels of the skin, joints, gastrointestinal (GI) tract, and kidneys in children. The main clinical features are non-thrombocytopenic purpura, GI manifestations, arthritis, and renal involvement. Most HSP cases have a sound outcome, with renal involvement being the most key prognostic factor influencing morbidity and mortality (1). This is especially true for severe kidney disease (SKD), as severe renal involvement is related to chronic renal impairment, nephritic or nephrotic syndrome, renal insufficiency, and renal failure. Therefore, the predictors for severe kidney disease have been a focus, and the risk factors at the time of diagnosis may aid in disease management. However, a standard and effective method to identify the risk factors for renal involvement is still lacking. Many studies focus on the prognostic factors of developing renal involvement in HSP, without consistent results (2–7). Some studies are not cohort studies, and some studies have a limited sample size. In addition, some studies targeting the Chinese population were restricted by a small sample size, the neglect of socioeconomic factors, and other initial changes of this disease, like HSP triggers and central nervous system (CNS) involvement (8). Considering these limitations of previous studies, the children with HSP were prospectively assessed to better identify the demographic findings and risk factors for SKD, thereby facilitating the better management of the disease. This prospective study aimed to explore risk factors of SKD within 3, 6, 12, and 24 months after the HSP diagnosis by analyzing the follow-up medical records within the same aforementioned timeframe.

The nomogram shows an easy and practical graphical tool to predict the risk assessments of a disease or death of patients and provide references for treatment, follow-up, and prognosis. At present, the nomogram has not been applied to the prediction of SKD.

Therefore, our goal was to build a prognostic model of 6-, 12-, and 24-month SKD outcomes in children with HSP, combining socioeconomic factors, clinical features, and treatments of this disease based on a large dataset gathered from high-volume institutions. So we analyzed two large prospective cohorts (development and validation cohorts) of patients with HSP to assess the performance of a nomogram to predict SKD status. The predictive value was determined according to discrimination, calibration, and clinical utility in separate internal and external validation cohorts.

This research was approved by the Medical Ethical Committee of the West China Second University Hospital of Sichuan University for Children and followed the Transparent Reporting of a Multivariable Prediction Model for Individual Prognosis or Diagnosis (TRIPOD) statement on reporting predictive models. As a result, we show an easy and practical graphic tool that maybe expand the clinical use of the nomogram in HSP.

## Methods

### Patient selection

**Figure 1:**
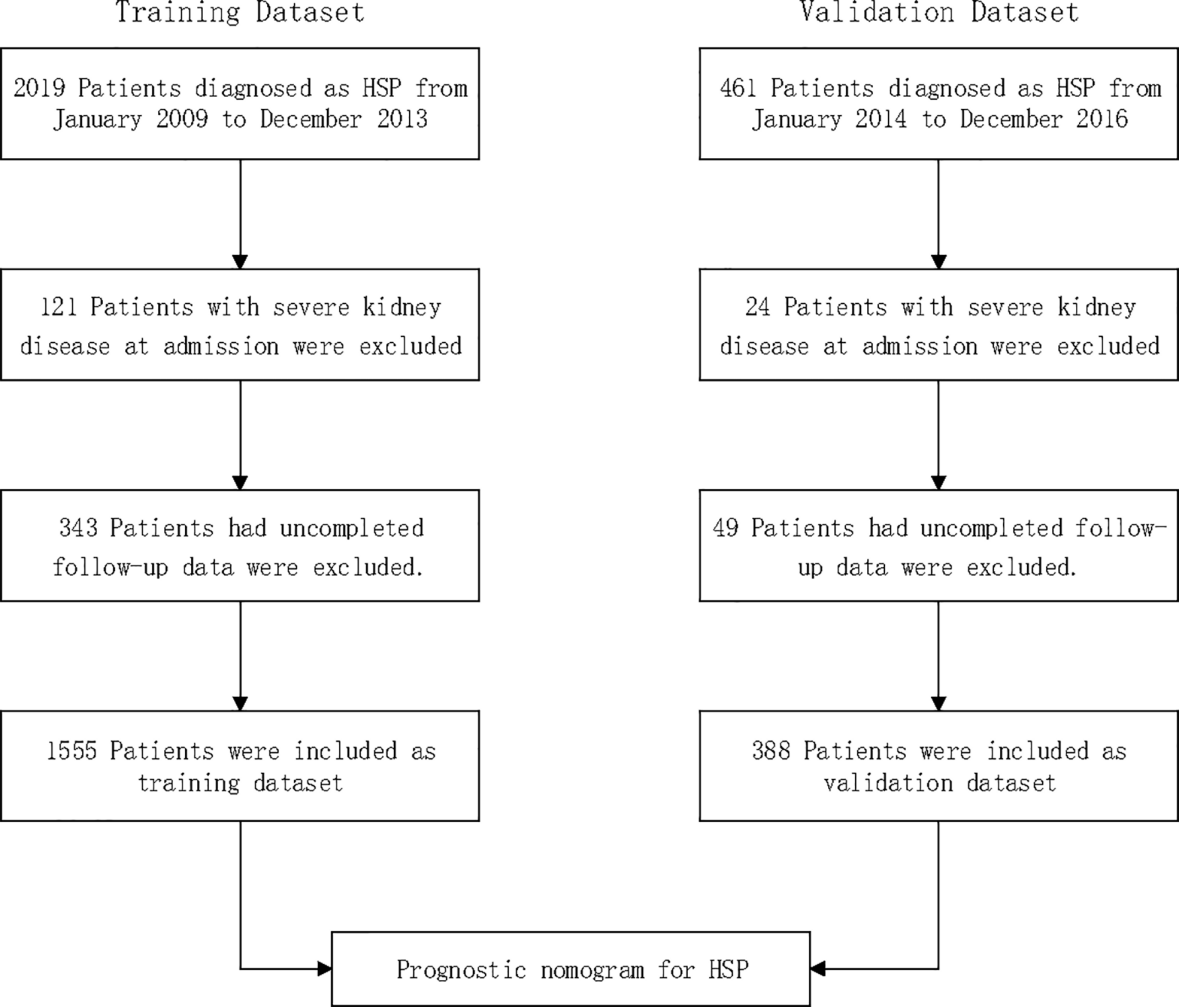
Study cohort.

A total of 2,480 consecutive patients aged 1 to 18 years diagnosed with HSP between 2009 and 2016 at a local hospital were considered for inclusion; 145 (5.8%) patients with severe kidney disease at admission were excluded; 392 (15.8%) had uncompleted follow-up data and were also excluded. Thus, 1,943 patients with HSP were included in total: 1555 patients included for the training dataset and 388 patients for the validation dataset (**Figure 1**).

### Collection of demographic, clinicopathological, and follow-up data

Patients were recruited on arrival at the pediatric inpatient department by the attending physician or by the physician at the ward. The following data were obtained from the hospital database: age at onset, gender, residence, onset season, exposure to known HSP triggers, skin manifestations (none, mild skin rash, severe skin rash, and digestive tract purpura), GI manifestations (none, mild symptom, and severe symptom), recurrent symptoms (yes *vs.* no), hormone therapy (not used, low dose, and high dose), renal involvement on admission (yes *vs.*no), arthritis, CNS involvement, angioedema (yes *vs.* no), and blood purification treatment (yes *vs.* no).

Skin involvement was graded as mild skin rash, severe skin rash, and digestive tract purpura. Mild skin rash appears only in the four limbs. Severe skin rashes are observed on both four limbs and the torso. Severe rashes included severe skin rashes and/or digestive tract purpura diagnosed by endoscopy. Patients with abdominal pain and gastrointestinal bleeding, diagnosed by occult-positive or gross bloody stools, were divided into severe GI manifestation groups. Patients with only abdominal pain were classified into the mild GI manifestation group. Arthritis was diagnosed by swelling or pain in the joints and surrounding soft tissue. Symptoms including headache, seizure, and coma were classified as CNS involvement. Recurrence was considered when a patient who was previously diagnosed as having HSP and who had been asymptomatic for at least 4 weeks presented with a new episode of illness (9). Hospitalized HSP patients were administrated hormone therapy (prednisone or methylprednisolone) when they were experiencing any of the following symptoms: gastrointestinal bleeding, angioneurotic edema, severe arthritis, and severe HSP nephritis according to the relevant guidelines of China (10).

Patients with HSP are defined as dependent on the EULAR/PRINTO/PRES-endorsed consensus criteria for HSP (11, 12). Renal involvement was determined by macroscopic or microscopic hematuria (more than three red blood cells in centrifuged urine specimen observed under the field of a high-magnification microscope) regardless of the presence of protein (2, 13).

Patients with macroscopic hematuria, proteinuria, and nephrotic syndrome with/without acute kidney injury were considered as severe kidney disease. In our study, proteinuria was defined as protein >0.15 g/day (3). Nephrotic syndrome was defined as massive proteinuria > 40 mg/m^2^/h or 50 mg/kg/24 h or a random sample of urinary protein-to-creatinine ratio exceeding 2.0, which caused severe hypoalbuminemia (<2.5 g/dl) (14).

Regular medical follow-up data were gotten *via* telephone, outpatient, internet, and other interaction tools. Patients were followed up for 24 months in development/training and validation cohorts. The data were gathered at multiple time points after HSP (at 3, 6, 12, and 24 months) covering severe kidney disease as the severe outcome after HSP; data obtained at 3, 6, 12, and 24 months after HSP were assessed as the outcomes.

### Selection of features

The least absolute shrinkage and selection operator (LASSO) Cox regression algorithm was employed for feature selection. The LASSO method was recruited to select the most useful predictive features from the original data set. The minimum tuning parameter (λ) for the LASSO Cox regression was selected *via* cross-validation.

### Statistical analysis

The Wilcoxon–Mann–Whitney test or Fisher’s exact test was recruited to analyze the differences in the distribution of variables in the development cohort and between the development and validation cohorts. A two-sided *p* < 0.05 was considered statistically significant. The predictive model of SKD with risk factors depicted in a nomogram was promoted through the rms package in R, version 3.6.2 (http://www.r-project.org/). Nomogram was built based on the probability of the occurrence of SKD using multivariate Cox regression in the development cohort and further proven in the external cohort and by using the rms package of R, version 3.6.2. Nomogram performance was determined in the development cohort with the calibration plot as an indicator of internal calibration. The Hosmer–Lemeshow test was used to analyze the goodness of fit, with Harrell’s C statistic for discriminative ability determination. Nomogram performance was analyzed in the validation cohort with the same methods as the development cohort in each follow-up time.

## Results

### Baseline characteristics of patients and outcomes

The clinical characteristics of development and validation cohorts are summarized in **Table 1**. The cohorts were similar in all of the clinical characteristics. During the two years of the follow-up period, 291 (14.98%) cases showed SKD in all datasets. Among cases with renal involvement at hospital admission, 183 (34.66%) cases developed into SKD. Among cases without renal involvement at hospital admission, 113 (7.99%) cases developed into SKD. Furthermore, the SKD rate in the training dataset after 3, 6, 12, and 24 months was 13.6%, 14.5%, 14.9%, and 15.2%, respectively. However, 60 cases in the validation dataset showed SKD. The SKD rate in the validation dataset after 3, 6, 12, and 24 months was 14.4%, 15.0%, 15.5%, and 15.5%, respectively; 96% of severe kidney disease occurred within the first 6 months of follow-up in both the training and validation datasets.

**Table 1:** Clinical and characteristics of development and validation cohort patients undergoing HSP.

Characteristic	Cohort, no. (%)		*p*
	Development (n = 1,555)	Validation (n = 388)	
**Age at onset**			0.3846
<6	398 (25.59)	91 (23.45)	
≥6	1,157 (74.41)	297 (76.55)	
**Gender**			0.8131
Boys	840 (54.02)	207 (53.35)	
Girls	715 (45.98)	181 (46.65)	
**Interval between symptom onset and diagnosis, days**			0.2308
0–3	797 (54.38)	211 (51.25)	
4–7	419 (22.68)	88 (26.95)	
≥8	339 (22.94)	89 (21.80)	
**Residence**			0.4469
Urban	626 (40.26)	148 (38.14)	
Rural	929 (59.74)	240 (61.86)	
**Onset season**			0.5211
Spring	348 (22.38)	97 (25.00)	
Summer	338 (21.74)	83 (21.39)	
Autumn	326 (20.96)	86 (22.16)	
Winter	543 (34.92)	122 (31.44)	
**Exposure to known HSP triggers**			0.6590
No	841 (54.08)	205 (52.84)	
Yes	714 (45.92)	183 (47.16)	
**Skin manifestations**			0.4241
Mild rash	1,041 (66.95)	268 (69.07)	
Serious skin rash or abdominal purpura	514 (33.05)	120 (30.93)	
**GI manifestations**			0.5095
No or Mild symptom	1,140 (73.31)	278 (71.65)	
Severe symptom	415 (26.69)	110 (28.35)	
**Arthritis**			0.3017
No	839 (53.95)	198 (51.03)	
Yes	716 (46.05)	190 (48.97)	
**Recurrent symptoms**			0.1297
No	1,489 (95.76)	378 (97.42)	
Yes	66 (4.24)	10 (2.58)	
**Angioedema**			0.1251
No	1,173 (75.43)	278 (71.65)	
Yes	382 (24.57)	110 (28.35)	
**CNS involvement**			0.1253
No	1,520 (97.75)	384 (98.97)	
Yes	35 (2.25)	4 (1.03)	
**Blood purification treatment**			0.3082
Not used	1,005 (64.63)	240 (61.86)	
Used	550 (35.37)	148 (38.14)	
**Hormone therapy**			0.8054
Not used	45 (2.89)	9 (2.32)	
Low dose	1,183 (76.08)	299 (77.06)	
High dose	327 (21.03)	80 (20.62)	
**Renal involvement**			0.4779
No	1,138 (73.18)	277 (71.39)	
Yes	417 (26.82)	111 (28.61)	

CNS, central nervous system; HSP, Henoch–Schönlein purpura; GI, gastrointestinal.

### Selection and design of prognostic predictors

**Figure 2:**
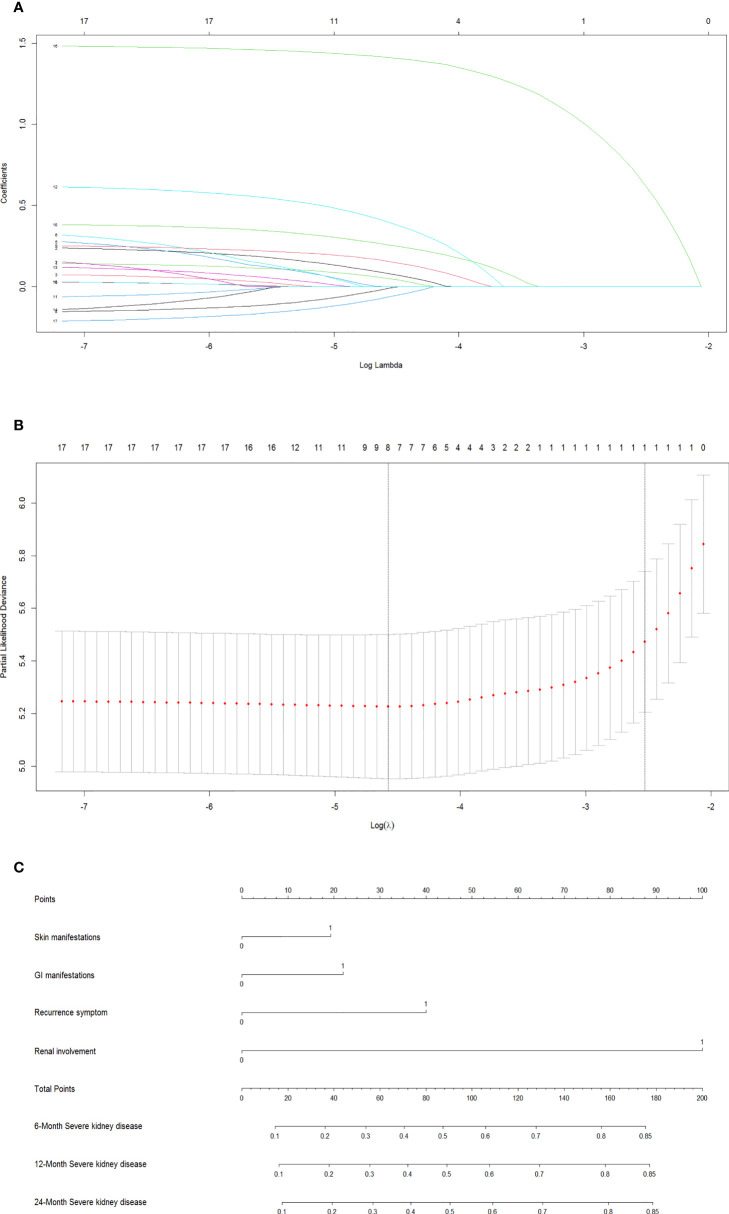
Screen the influencing factors using LASSO regression and estimate probability of SKD by nomogram. The punishment process of the model **(A)**. The change process of the best punishment coefficient λ in LASSO regression model **(B)**. Nomogram to estimate probability of SKD at 6, 12, and 24 months **(C)**. LASSO, least absolute shrinkage and selection operator; SKD, severe kidney disease.

In the training dataset, the LASSO Cox regression algorithm was recruited to determine the most significant predictors and construct the prognostic nomogram. Thirteen clinical features were employed in the LASSO Cox regression for 1,000 bootstrap iterations, and right features with non-zero coefficients were then selected with a minimum lambda value of 0.0103 (**Figure 2**). This algorithm ultimately included eight variables: age at onset, residence, exposure to known HSP triggers, skin manifestations, GI manifestations, recurrent symptoms, renal involvement, and degree of hormone therapy.

### Development of a multivariate prognostic nomogram

The results of the final multivariate Cox model that incorporated eight independent variables (according to the results above) are shown in **Table 2**. Multivariate analysis showed that odds ratio (95% CI), serious skin rash or digestive tract purpura (1.36 [1.05–1.77]), severe GI manifestations (1.46 [1.12–1.90]), recurrent symptoms (2.03 [1.27–3.25]), and renal involvement (4.93 [3.77–6.45]) were independently correlated with SKD. These independently associated risk factors were recruited to build a simple-to-use SKD risk estimation nomogram (**Figure 2**).

**Table 2:** Multivariate Cox regression analysis in training dataset.

Variable	HR (95%)	Coefficient	*p-*Value
**Age at onset**			
<6	Ref		
≥6	1.23 (0.87, 1.74)	0.20792	0.2367
**Residence**			
Urban	Ref		
Rural	1.21 (0.92, 1.59)	0.18915	0.1736
**Exposure to known HSP triggers**			
No	Ref		
Yes	0.85 (0.65, 1.10)	−0.16729	0.2076
**Skin manifestations**			
No or mild rash	Ref		
Serious skin rash or digestive tract purpura	1.36 (1.05, 1.77)	0.30918	0.0209*
**GI manifestations**			
No or mild symptom	Ref		
Severe symptom	1.46 (1.12, 1.90)	0.37715	0.0055*
**Recurrent symptoms**			
No	Ref		
Yes	2.03 (1.27, 3.25)	0.70663	0.0033*
**Hormone therapy**			
Not used	Ref		
Low dose	2.28 (0.73, 7.15)	0.82565	0.1565
High dose	1.43 (0.43, 4.69)	0.35441	0.5594
**Renal involvement**			
No	Ref		
Yes	4.93 (3.77, 6.45)	1.59592	<0.0001*

GI, gastrointestinal.

*p-value < 0.05.

### Internal and external data validation/validation of the nomogram

**Figure 3:**
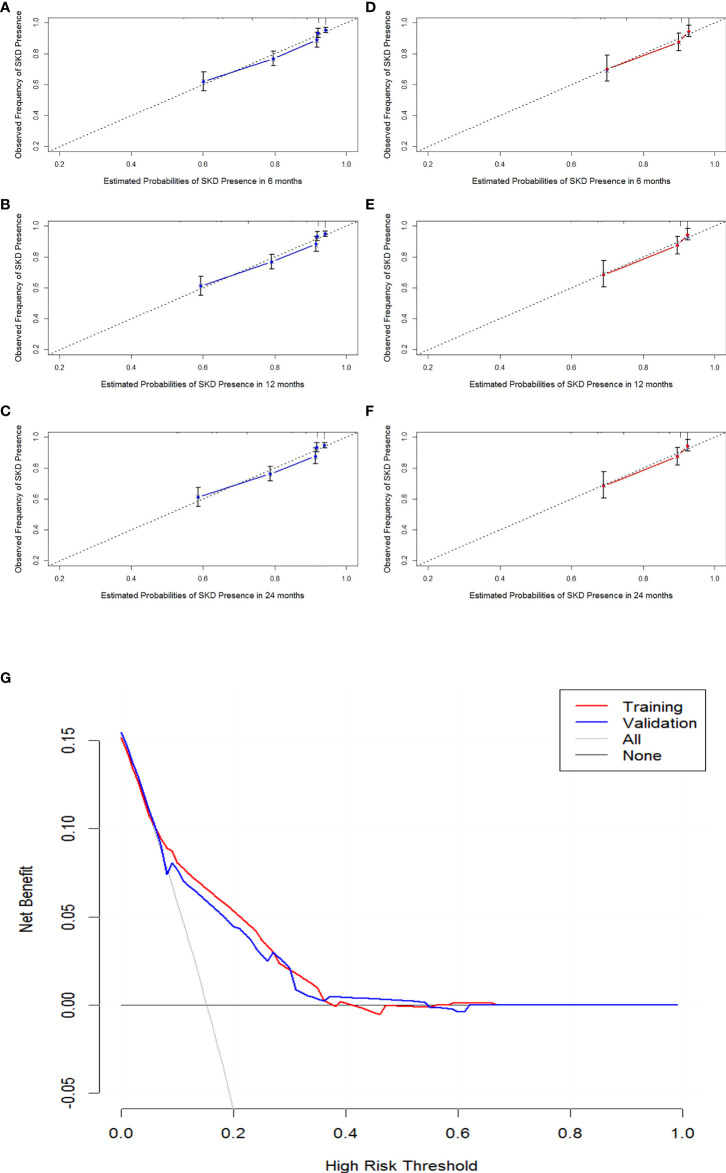
Calibration curves of 6-month, 1-year, and 2-year SKD and the decision curve analysis (DCA) for the nomogram. Calibration curve of the nomogram in the training cohort **(A–C)**. Calibration curve of the nomogram in the validation cohort **(D–F)**. The DCA for the nomogram in training and validation dataset **(G)**. The gray line represents the assumption that all patients have SKD. Thin black line (parallel to the x-axis) indicates that no patients have SKD. The blue line represents prediction nomogram scheme in validation dataset. The red line represents prediction nomogram scheme in training dataset. SKD, severe kidney disease.

The internal validation, external data validation, and calibration of the nomogram were conducted using a 1,000 bootstrap analysis. The training dataset was used to make an internal validation. The C-index was 0.751 (95% CI, 0.734–0.769) for the prognostic nomogram and 0.742 with bootstrap resampling. In contrast, the validation dataset was used to conduct external validation. The C-index was 0.714 (95% CI, 0.678–0.750) for the prognostic nomogram and 0.715 with bootstrap resampling. Moreover, the calibration curve showed sound agreement between the predicted and actual probability of SKD in both the training and validation datasets (**Figures 3A–F**).

### Clinical utility and validity of the nomogram

The decision curve analysis for the nomogram suggested that the nomogram was feasible for making valuable and informed judgments of the prognosis (**Figure 3**). Our results suggested that if the threshold probability of predicting SKD in patients was approximately 30%–50% based on this nomogram, using this nomogram to guide treatment for HSP patients would provide more benefits than the “treat all patients” and the “treat no patient” schemes.

## Discussion

We developed and validated, for the first time, clinical feature-based nomograms for the individualized prediction of SKD in patients with HSP. The nomogram incorporates four items of clinical features: serious skin rash or digestive tract purpura, severe GI manifestations, recurrent symptoms, and renal involvement. Model performance, as reflected by the C-statistic, was 0.751 (95% CI, 0.734–0.769) in the training dataset and 0.714 (95% CI, 0.678–0.750) in the validation dataset. It demonstrates that the relatively simple nomogram provides a good prediction of outcomes in prospective study-based data.

Our cohort is representative of pediatric HSP patients. The SKD prevalence of 14.98% in the present cohort of HSP patients in children is in line with previously reported rates of 15% in studies with at least 2 years of follow-up (15, 16). In this study, 85.19% of SKD occurred during the 3 months of follow-up, whereas 97.22% occurred during the 6 months of follow-up, which is in line with previously published data. A previous study also conducted 6 to 12 months’ follow-ups (17). KDIGO (Kidney Disease: Improving Global Outcomes) guideline suggests that urinary monitoring is necessary for 6 months (18). Our follow-up data are supportive of the suggestion that a 6-month follow-up is a more prudent method for disease evaluation and amelioration (19).

We found that severe GI manifestations are a significant predictor for HSP nephritis in our Cox regression analysis, regardless of renal involvement symptom (test for protein and/or blood in urine) for patients at hospital admission. It implied that patients with severe GI manifestations have a higher risk of developing SKD, even though they may have normal urine test results. A recent study in China found that newly enrolled patients with GI were more likely to develop renal involvement during follow-up (20). Another study using machine learning prediction found similar results (21). Thus, patients with normal urine rest require 6 months of follow-up (22). Pediatric patients with severe GI symptoms, such as abdominal pain with GI bleeding and colic, require special attention in monitoring nephritis-related symptoms (2, 6, 23).

We found that the severe skin rash/digestive tract purpura and recurrent symptoms were strongly predictive of SKD. The risk factors for developing nephropathy in the course of HSP, according to a meta-analysis carried out by Chan et al., included persistent purpura and the occurrence of relapses (23). It was consistent with another study that reported that persistent skin rash for more than 1 month was a crucial risk factor for HSP patients to develop nephropathy and recurrence (24). Another group argued that patients presenting with a recurrent episode are supposed to receive renal screening again regardless of previous normal findings and followed up to ensure complete remission (25). Jiang *et al.* and Cao *el al.* found that relapse was an independent risk factor of HSP nephritis (HSPN) or renal damage (20, 21). Thus, early-onset HSP patients with severe skin symptoms and recurrence should receive special care.

We found that 34.66% of patients with renal involvement developed SKD in a 2-year follow-up. In Cox regression analysis, renal involvement at hospital admission is a risk factor for SKD. Other studies found that the severity of renal involvement at HSP onset determines the severity of nephritis and long-term HSP prognosis (15, 26). Thus, patients with renal involvement, especially those with protein in the urine, require close monitoring of urine since disease onset (3).

In our study, 97.11% of hospitalized cases underwent hormone treatment. However, we did not find a significant correlation between hormone treatment with disease prognosis in our Cox regression analysis. Obviously, clinical symptoms have more predictive power in prognosis. Other studies and reviews also mentioned that there is no evidence supporting a preventive role of hormone treatment during HSP onset in nephritis progression (4, 17). A systemic evaluation implicated that steroids could not provide protection against complications and should not be used as a preventive measure (19).

Here, age at onset was not a predictor of poor prognosis in Cox multivariate analysis, even if our previous and others’ studies showed that older children (≥6 years old) are more likely to have the more severe disease at presentation (5–7, 20, 27–30).

However, the present study has several limitations. First, the accuracy of our nomogram was determined through both internal and external validation, but the nomogram was developed and externally validated based on two prospective cohorts from one medical institution; therefore, potential bias was inevitable. A multicenter trial would allow further evaluation of the accuracy and applicability of this novel nomogram in a wider population of patients with HSP. Second, there was attrition in follow-up between 1 and 24 months, which might due to the difficulty of extensive follow-up for patients with overall good outcomes to be involved in extensive follow-up; thus, this might cause a bias toward good prognosis in the patients lost to follow-up. However, our dropout rate was similar to that of other studies. Thus, we established a reliable predictive model, providing vital results to interpret the long-term outcome in this patient population.

In summary, our nomogram study reveals four clinical features as risk factors for SKD: serious skin rash or digestive tract purpura, severe GI manifestations, recurrent symptoms, and renal involvement. Specific clinical interventions to these clinical features may provide a positive influence in preventing SKD in HSP patients (23). However, due to the limitations in this study described above, caution should be taken in the interpretation of our results.

## Data availability statement

The raw data supporting the conclusions of this article will be made available by the authors, without undue reservation.

## Ethics statement

The studies involving human participants were reviewed and approved by West China Second University Hospital. Written informed consent for participation was not required for this study in accordance with the national legislation and the institutional requirements.

## Author contributions

KW and L-QD contributed to the study design. S-LJ, LL, YC, and XP supervised the data collection. Data analysis was performed by KW. KW and X-MS wrote the main manuscript text. L-QD and L-NQ reviewed and edited the manuscript. All authors contributed to the article and approved the submitted version.

## Funding

This research was supported by funding from The Central Government Funds of Guiding Local Scientific and Technological Development for Sichuan Province of China (No. 2021ZYD0105), the Sichuan Province Key Research and Development Program (No. 22ZDZX0014) and Fundamental Research Funds for the Central Universities (SCU2021E4251).

## Acknowledgments

We would like to thank Wen Deng for proofreading our manuscript.

## Conflict of interest

The authors declare that the research was conducted in the absence of any commercial or financial relationships that could be construed as a potential conflict of interest.

## Publisher’s note

All claims expressed in this article are solely those of the authors and do not necessarily represent those of their affiliated organizations, or those of the publisher, the editors and the reviewers. Any product that may be evaluated in this article, or claim that may be made by its manufacturer, is not guaranteed or endorsed by the publisher.
